# cPCN-Regulated SnO_2_ Composites Enables Perovskite Solar Cell with Efficiency Beyond 23%

**DOI:** 10.1007/s40820-021-00636-0

**Published:** 2021-04-01

**Authors:** Zicheng Li, Yifeng Gao, Zhihao Zhang, Qiu Xiong, Longhui Deng, Xiaochun Li, Qin Zhou, Yuanxing Fang, Peng Gao

**Affiliations:** 1 CAS Key Laboratory of Design and Assembly of Functional Nanostructures, and Fujian Provincial Key Laboratory of Nanomaterials, Fujian Institute of Research on the Structure of Matter, Chinese Academy of Sciences, Fuzhou, 350002 Fujian People’s Republic of China; 2 College of Chemistry, Fuzhou University, Fuzhou, 350116 People’s Republic of China; 3 Laboratory for Advanced Functional Materials, Xiamen Institute of Rare Earth Materials, Haixi Institute, Chinese Academy of Sciences, Xiamen, 361021 People’s Republic of China

**Keywords:** Electron transport layer, Perovskite solar cell, Carbon nitride, SnO_2_

## Abstract

Efficient electron transport layers (ETLs) not only play a crucial role in promoting carrier separation and electron extraction in perovskite solar cells (PSCs) but also significantly affect the process of nucleation and growth of the perovskite layer. Herein, crystalline polymeric carbon nitrides (cPCN) are introduced to regulate the electronic properties of SnO_2_ nanocrystals, resulting in cPCN-composited SnO_2_ (SnO_2_-cPCN) ETLs with enhanced charge transport and perovskite layers with decreased grain boundaries. Firstly, SnO_2_-cPCN ETLs show three times higher electron mobility than pristine SnO_2_ while offering better energy level alignment with the perovskite layer. The SnO_2_-cPCN ETLs with decreased wettability endow the perovskite films with higher crystallinity by retarding the crystallization rate. In the end, the power conversion efficiency (PCE) of planar PSCs can be boosted to 23.17% with negligible hysteresis and a steady-state efficiency output of 21.98%, which is one of the highest PCEs for PSCs with modified SnO_2_ ETLs. SnO_2_-cPCN based devices also showed higher stability than pristine SnO_2_, maintaining 88% of the initial PCE after 2000 h of storage in the ambient environment (with controlled RH of 30% ± 5%) without encapsulation.

**Supplementary Information:**

The online version contains supplementary material available at 10.1007/s40820-021-00636-0.

## Highlights

- The (SnO_2_-cPCN) ETL shows superior electron mobility of 3.3 × 10^−3^ cm^2^ V^−1^ s^−1^, which is about three times higher than that of pristine SnO_2_.
- The less wettable SnO_2_-cPCN leads to perovskite layers with reduced grain boundaries and enhanced qualities due to suppressed heterogeneous nucleation of perovskite.
- The PSCs based on SnO_2_-cPCN showed negligible *J*–*V* hysteresis and two champion PCE of 23.17% and 20.3% on devices with 0.1 and 1 cm^2^ active area, respectively.

**Supplementary Information:**

The online version contains supplementary material available at 10.1007/s40820-021-00636-0.

## Graphical abstract

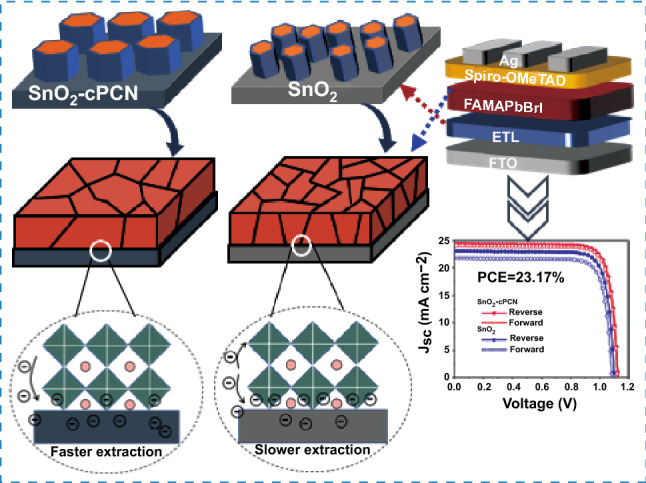

## Introduction

Organic–inorganic hybrid perovskite-based solar cells (PSCs) are considered the paradigm shift of traditional crystalline silicon photovoltaics due to their fascinating optoelectronic properties and easy solution processability [1–3]. Since the first report in 2009 [4, 5], the certified power conversion efficiency (PCE) of PSCs has exceeded 25%, encouraging the research community to explore further the general principles behind the material design, film formation, device structure, and operational mechanism [6–10]. In the early stage of PSC studies, mesoscopic titanium oxide (m-TiO_2_) was frequently used as the electron transport layer (ETL), facilitating the certified world record PCEs [11]. However, the high temperatures (> 500 °C) sintering process damaged the solution processability and limited the application of PSCs in flexible and tandem devices [12]. Besides, the m-TiO_2_ presents high photocatalytic activity under the illumination of ultraviolet light, undermining the long-term stability of PSCs [13]. In this regard, planar-type PSCs become potential alternatives to mesoporous types and growing high-quality ETL at low temperatures (≤ 150 °C) has been emphasized to obtain high-efficiency planar-type devices. Among all the candidate ETLs, low-temperature-processed SnO_2_ has demonstrated advantages such as high optical transmittance, congruous energy levels, robust chemical and UV stability, and balanced electron mobility (10^−4^ cm^2^ V^−1^ s^−1^) [14–16], enabling several scintillating PSCs with record-breaking performances simultaneously [17, 18].

However, charge accumulation may happen at the SnO_2_/perovskite interface resulting in severe hysteresis due to much lower electron mobility of SnO_2_ ETL than perovskite absorbers (0.5–30 cm^2^ V^−1^ s^−1^) [19–22]. To alleviate the hysteresis effect, dopants or additives such as metal cations and polymers were added to enhance the conductivity of SnO_2_ and hence the PCE of devices. Ren et al. investigated the effect of Nb-doping in SnO_2_ and decreased hysteresis due to the enhancement of electron mobility from 1.02 × 10^−4^ to 2.16 × 10^−4^ cm^2^ V^−1^ s^−1^ [23]. Wei et al. prepared a polymer-incorporated SnO_2_ colloidal ink to ameliorate the compactness and wetting property of the SnO_2_ layer, and suppressed hysteresis emerged due to better coverage of perovskite film on the SnO_2_-in-polymer matrix [24]. The results revealed that the underlayer's surface properties have a crucial influence on the morphology and quality of the perovskite films above.

Shreds of evidence have indicated that these grain boundaries might cause charge recombination ascribed to the presence of charge-trapping recombination centers, and tremendous efforts have been devoted to reducing the grain boundaries of perovskite films. Liu and Huang’s groups reported, respectively, that the incorporation of ethylene-diamine-tetra-acetic-acid (EDTA) or red-carbon quantum dots (RCQs) into SnO_2_ can increase the wettability at ETL/perovskite interface and generate high-quality perovskite films with enlarged grain size and reduced grain boundaries [15, 19]. In contrast, Huang et al. demonstrated that the non-wetting polymeric HTL surface could also lead to perovskite layers with large grains [25]. Given the discussions above, the surface conditions of the underlayer should be well controlled to generate high-quality and ideally defect-free (less) perovskite films. The influence of the wettability of the underlayer on the perovskite films needs to be further explored.

Targeting the conductivity of SnO_2_ and its influence on the quality of the perovskite layer, the presented work realized crystalline polymeric carbon nitride (cPCN)-composited SnO_2_ (SnO_2_-cPCN) ETL with superior electron mobility of 3.3 × 10^−3^ cm^2^ V^−1^ s^−1^, which is more than three times higher than that of pristine SnO_2_. The PSCs based on SnO_2_-cPCN exhibited negligible current density–voltage (*J–V*) hysteresis due to the decreased charge accumulation at the perovskite/ETL interface under the increased electron mobility. Besides, the SnO_2_-cPCN surface became smoother and less wettable. On this basis, the perovskite absorber layers with reduced grain boundaries and enhanced qualities were realized due to suppressed heterogeneous nucleation of perovskite. Incorporating cPCN into SnO_2_ not only enhanced the electron mobility of ETL via effectively filling the electron trap states but also affected the growth of perovskite grains, reducing non-radiative recombination. Finally, planar PSCs based on SnO_2_-cPCN presented a champion PCE of 23.17% on devices with a small active area (0.1 cm^2^) and a promising PCE of 20.3% on devices with a large active area (1 cm^2^).

## Experimental Section

### Materials and Reagents

SnO_2_ colloid precursor (tin(iv) oxide, 15% in H_2_O colloidal dispersion) was purchased from Alfa Aesar. The FAI and MABr were synthesized according to the procedures in previously reported methods. Lead iodide (99.99%) and lead bromide (99.99%) were bought from TCI. The spiro-MeOTAD were bought from Derthon; 4-tert-butylpyridine (96%), bis(trifluoromethane)sulfonimide lithium (LiTFSI) salt (99.95%), chlorobenzene (99.8%), 2N, N-dimethylformamide (99.8%), and dimethyl sulfoxide (99.9%) was purchased from Alfa Aesar.

### Preparation of cPCN and PSCs

#### Preparations of the g-CN, cPCN

The g-CN powder was synthesized by thermal condensation of the urea. Typically, melamine (8 g) was heated to 500 ºC for 4 h at a rate of 12 °C min^−1^ in a muffle furnace in an air atmosphere. The crystalline PCN was synthesized by an ionothermal approach. In detail, 1.2 g of melamine was mixed with KCl (6.6 g) and LiCl (5.4 g) in a glovebox, and the mixture was then heated to 550 ºC under an N_2_ atmosphere (2 L min^−1^) in a muffle furnace.

#### Preparations of the SnO_2_-cPCN Precursor

The synthesized cPCN NCs powder was first dispersed in deionized water, ultrasonicated for 10 h, and then filtered with a 0.45 mm filter to obtain cPCN NCs water solution of different concentrations. Then, the SnO_2_ colloid precursor (15 wt.%) was diluted with deionized water to the concentration of 2.5 wt.% and was stirred at room temperature for 2 h. The cPCN NCs and SnO_2_ solutions were mixed with a volume ratio of 1:1 ratio and then ultrasonicated for 1 h.

#### Device Fabrication

Chemically etched FTO glass substrates were cleaned with a detergent solution, deionized water, acetone, and anhydrous ethanol for 15 min, respectively. Next, the substrates were further cleaned with plasma treatment for 15 min. The SnO_2_ solution (Alfa) was spin-coated on the FTO substrates at 3000 rpm for 30 s, followed by annealing at 150 °C for 30 min. The SnO_2_-cPCN solution also underwent the same procedures. The substrate was then cool down to room temperature on a spin coater. The (FAPbI_3_)_0.9_(MAPbBr_3_)_0.1_ perovskite solution (PbI_2_, FAI, PbBr_2_, MACl, MABr, in DMF: DMSO = 9:1 volume ratio) was spin-coated at 1000 rpm for 10 s and 5000 rpm for 30 s onto the FTO/SnO_2_ substrate. 200 μL of chlorobenzene was dropped on the spinning substrate at 8 s before the program finish, and the FTO/SnO_2_/perovskite sample was heat-treated at 150 °C for 15 min. Then, the hole transporting layer was deposited on top of the perovskite layer at a spin rate of 4000 rpm for 20 s using a spiro-OMeTAD solution. For the spiro-OMeTAD solution, 72.3 mg of spiro-OMeTAD was dissolved in 1 mL of chlorobenzene with additives of 17.5 μL of bis(trifluoromethylsulfonyl)imide lithium salt (Li-TFSI, Sigma-Aldrich) solution (520 mg mL^−1^ in acetonitrile), 28.8 μL of 4-tert-butylpyridine (TBP, Sigma-Aldrich). Finally, 120 nm of the silver counter electrode was thermally evaporated under a high vacuum.

### Characterization

SEM measurements were performed using a SUPRA 55, Zeiss, Germany, operated at an acceleration voltage of 5 kV. The XRD patterns were measured using a SmartLab X-ray powder diffractometer with an angle range of 2θ = 3° to 60°. UV–vis absorption spectra were recorded on a spectrophotometer (Agilent Cary 5000) in the 350–850 nm wavelength range at room temperature. The steady-state PL spectra were obtained using a fluorescence spectrophotometer (FLS980, Edinburgh Instruments). Current density–voltage (*J-V*) characteristics were measured using a source meter (Keithley 2400) under 100 mW cm^−2^ simulated AM 1.5 G irradiation with a solar simulator (Enli Tech, Taiwan) by reverse (1.2 to -0.1 V) scans or forward scan (from −0.1 to 1.2 V) modes at a scan speed of 200 mV s^−1^. The hysteresis indices (H_hysteresis_) of the devices are calculated based on Eq. 1.

1 $${\text{H}}_{{{\text{hysteresis}}}} = \frac{{{\text{PCE}}_{{{\text{reverse}}}} - {\text{PCE}}_{{{\text{forward}}}} }}{{{\text{PCE}}_{{{\text{reverse}}}} }} \times 100\%$$

The active area of devices was defined by a metal shadow mask of 0.1 or 1 cm^2^. The dark current–voltage curves were recorded with an electrochemical workstation (Zennium Zahner, Germany). In detail, the measurement works as follows: For the first edge potential of -1.0 V, the second edge potential of 1.5 V was applied. The EQE was characterized by the QE-R systems (Enli Tech.), and the measurement scope was 300–900 nm. EIS measurements were carried out in the dark at 0.5 V applied voltage using an electrochemical workstation (Zennium Zahner, Germany) with an AC perturbation of 10 mV ranging from 100 to 1 MHz at room temperature with 60% humidity (Fig. S6). FTIR spectra were recorded on a Nicolet iS 50 Spectrometer. The roughness of the films was recorded using atomic force microscopy (AFM, Multimode-8J, America). The water contact angle was measured at a Data physics OCA-20 drop shape analyzer.

To gain insights into the charge transport, we measured electron mobility using different ETLs in the same device structure. Specifically, the electron-only device was designed and fabricated using FTO/ETL/PCBM/Ag structure, as shown in the inset in Fig. 2b (later). In this analysis, we assumed that the current is only related to electrons. When the effects of diffusion and the electric field are neglected, the current density can be determined by the SCLC [26]. The different ETLs were spin-coated on FTO. Then, 120-nm-thick Ag was deposited on FTO/ETL/PCBM samples. The dark J–V curves of the devices were performed on a Keithley 2400 source at ambient conditions. The electron mobility (*μ*_*e*_) is extracted by fitting the *J–V* curves using the Mott–Gurney law (Eq. 2):

2 $$\mu_{e} { = }\frac{{{\text{8JL}}^{{3}} }}{{{9}\varepsilon \varepsilon_{{0}} {(}V_{{{\text{app}}}} - V_{r} - V_{bi} )^{{2}} }},$$

where *J* is the current density, *L* the thickness of different ETLs, *ε*_0_ the vacuum permittivity, ε_r_ the dielectric permittivity of various ETLs, *V*_app_ the applied voltage, *V*_*r*_ the voltage loss due to radiative recombination, and *V*_*bi*_ the built-in voltage owing to the different work function between the anode and cathode.

## Results and Discussion

### Crystalline Polymeric Carbon Nitride Characterization

Carbon derivatives, such as carbon nanotubes, fullerene, and graphene (oxide), have been widely used as additives or interlayers in PSCs [27, 28]. Particularly, traditional graphitic carbon nitride (g-CN) was reported to facilitate high-efficiency PSCs. For example, Jiang et al. added g-CN in precursor solutions to improve the quality and conductivity of perovskite films [29]. Similarly, Li et al. developed a series of functionalized g-CN (SO_3_–, OH–, NH_3_–, or NO_3_–C_3_N_4_) to modify the perovskite precursor solution and achieved the best PCE of 20.08% with NO_3_-C_3_N_4_ [30]. Recently, Chen et al. used hybrid quantum dots of SnO_2_/g-CN was used as the ETL in PSCs and demonstrated that the g-CN could reform the electronic density distribution around the neighboring SnO_2_ crystal unit to effectively eliminate the oxygen-vacancy type trap centers and promote the electron transport [31]. Compared with the widely used g-CN, the cPCN is the highly crystalline counterpart with fully conjugated π-electron systems and higher conductivity [32, 33], which may further enhance the functionality of SnO_2_ based ETLs. It is worth noting that traditional poly-heptazines are often (and misleadingly) classed as 'graphitic carbon nitride,' 'graphitic CN' or 'g-C_3_N_4_′ in literature, including our own reports [33]. To that end, we have proposed to correct the term of ‘gCN’ to ‘PCN’ in one of our review papers [32].

**Fig. 1:**
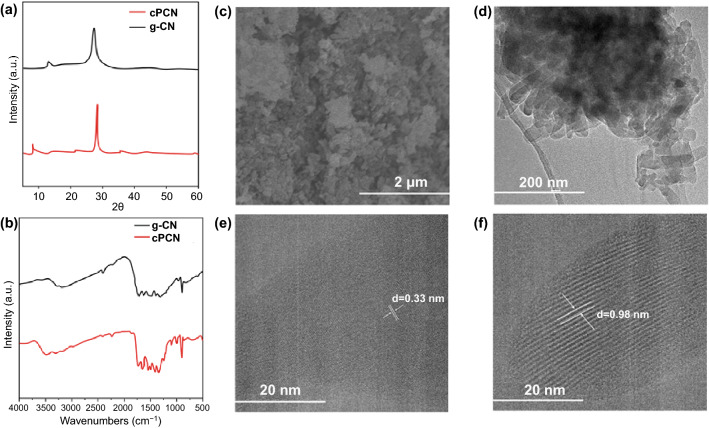
**a**, **b** XRD patterns and FTIR spectra of cPCN and g-CN. **c** SEM images and **d** TEM images of cPCN. **e, f** HRTEM images for cPCN

The preparation protocol of the g-CN and cPCN semiconductor is described in the experimental section. In this study, preheated melamine was used as a precursor in the presence of KCl/LiCl to synthesize cPCN with tri-s-triazine subunits. The high crystallinity nature of cPCN was verified by X-ray diffraction (XRD), as illustrated in Fig. 1a. The cPCN exhibited a narrower and shifted diffraction peak at 28.3° with a full width at half-maximum (FWHM) compared to traditional g-CN (27.4°), indicating the well-developed and condensed crystal structure with enhanced interaction between layers [33]. The peak at 13.0° corresponding to the inter-plane distance of 0.618 nm for g-CN is shifted to 8.0° with a repeat unit of 1.099 nm due to an unfolded in-plane network associated with sufficient condensation of the conjugated framework (Fig. S1) [33]. The FTIR spectra in Fig. 1b further exhibit the structure information of cPCN and g-CN. The broad peaks between 3500 and 3000 cm^−1^ stem from the terminal amino groups, while the peak at 2150 cm^−1^ originates from terminal cyano groups (C≡N) owing to the loss of ammonia on the surface of traditional g-CN [34]. The set of peaks between 1700 and 900 cm^−1^ are characteristic signals from tri-s-triazine derivatives [35].

The as-prepared cPCN sample exhibits porous structures under scanning electron microscopy (SEM) (Fig. 1c). The transmission electron microscopy (TEM) image presents stacked layers of nanosheets (Fig. 1d). The high-resolution TEM images of cPCN are illustrated in Fig. 1e, f, revealing a clear hexagonal lattice structure with two lattice fringes. The lattice fringe of 0.33 nm may be assigned to the interlayer distance, while the 0.98 nm lattice fringe is likely originated from the in-plane periodicity. Furthermore, more information and discussions about the difference between PCN and cPCN, including morphology, structure, optical properties, electrical property, and stability, can be found in one of our works [33].

### Fabrication and Characterization of SnO_2_-cPCN Film

We plotted XRD patterns of SnO_2_ and SnO_2_-cPCN based films to investigate the structural properties and phase composition (Fig. S2). These peaks match well with characteristic diffractions of SnO_2_ (JCPDS No. 01-077-0452). Except the signals from the underlying FTO, no obvious peak relating to cPCN, SnO_2_, or any other impurity is detected. It is important to note that due to the very thin nature of the deposited layers (ca. 30 nm), minority phases may be challenging to be identified.

**Fig. 2:**
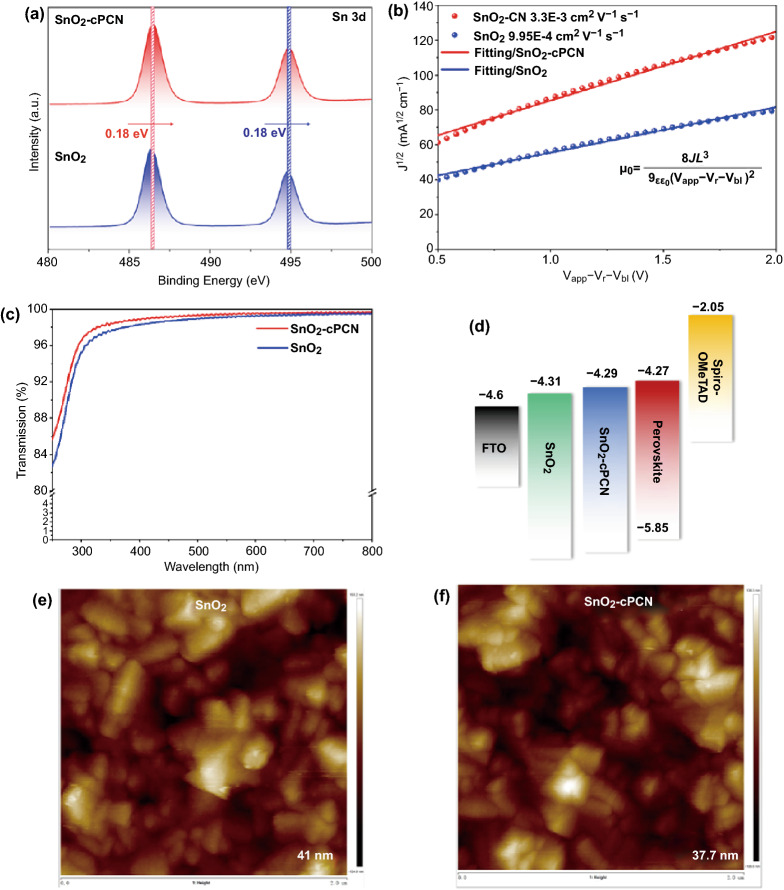
Characterization of SnO_2_ and SnO_2_-cPCN ETLs. **a** XPS spectra of films deposited on quartz substrates. **b** Electron mobility calculation using the SCLC model with the device structure of FTO/ETL/PCBM/Ag. **c** Optical transmission spectra on quartz substrates. **d** Possible band alignment of the ETLs and perovskite layer according to the UPS measurements. **e**, **f** AFM topographical images of SnO_2_, and SnO_2_-cPCN films

Based on the previous studies, the novel cPCN with higher crystallinity and conductivity may have the potential to improve the performance of SnO_2_ ETL. Specifically, X-ray photoelectron spectra (XPS) of SnO_2_ and SnO_2_-cPCN films deposited on quartz substrates are conducted (Figs. 2a and S3) to explore the interactions between cPCN and SnO_2_. We calibrated the binding energy scale for all XPS measurements to the carbon 1 s line at 284.8 eV. After the cPCN incorporation, the binding energies of Sn 3d_3/2_ and Sn 3d_5/2_ at 486.34 and 494.77 eV shift slightly to higher values at 486.52 and 494.87 eV, respectively. The blue shift (toward higher binding energy) of Sn 3d signals indicates the electron transfer and may promote electron mobility [36–39]. Besides, the SnO_2_-cPCN film presents two additional peaks at 400.08 and 404.16 eV originating from the N1s of cPCN (Fig. S3a, b). Consequently, the cPCN is successfully composited and chemically bonded with SnO_2_.

The speculated enhancement in electron mobility is further confirmed by measuring the *J–V* characteristics of electron-only devices with a structure of FTO/ETL/PCBM/Ag based on SnO_2_-cPCN and pristine SnO_2_. Unless stated otherwise, all the characterizations are based on SnO_2_-cPCN films fabricated by adding 0.6 mg mL^−1^ cPCN solution to the colloidal solution of SnO_2_. The optimization process toward the highest PSC performance was recorded in the device fabrication section. As illustrated in Fig. 2b, the electron mobility of these two ETLs can be calculated using the space charge-limited current (SCLC) model fitted by the Mott–Gurney law [40, 41]. The electron mobility of SnO_2_-cPCN was 3.3 × 10^−3^ cm^2^ V^−1^ s^−1^, which was one magnitude larger than those of pristine SnO_2_ (9.95 × 10^−4^ cm^2^ V^−1^ s^−1^). This is consistent with the conclusion of the XPS test. Considering that the carbon and nitrogen atoms of cPCN are *sp*^2^-hybridized to form fully π-conjugated electronic structures, we speculate that the excess electron traps in SnO_2_ could be consumed under the electron-rich conditions [27], which consequently increased the mobility and decreased the work function [36]. This, in turn, led to different way of band-bending and resulted in a decrease of the energy barrier at the SnO_2_/perovskite interface [36, 42], which can significantly affect charge extraction, collection, and recombination in PSC. The higher electron mobility can effectively facilitate electron transfer in the PSCs, reduce charge accumulation at the ETL/perovskite interface, improve efficiency, and suppress hysteresis in the PSCs [43]. Additionally, the optical transmission spectra of SnO_2_ and SnO_2_-cPCN films coated on quartz substrates are presented in Fig. 2c. Both samples exhibit high average transmittance in the visible region, demonstrating excellent optical quality to pledge that most light can pass through and be absorbed by the perovskite layer.

Ultraviolet photoelectron spectroscopy (UPS) measurements are performed to estimate band position shifts after hybridizing cPCN in SnO_2_ (Fig. S4a). According to the formula of work function (WF) = 21.22 eV − *E*_cutoff_ (cutoff binding energy), the WF of SnO_2_-cPCN is calculated to be − 4.32 eV. Then, the E_VBM_ of SnO_2_-cPCN can be calculated to be − 8.08 eV by *E*_VBM_ = WF—*E*_F, edge_ (Fermi edge). It was reported that the WF, conduction band (*E*_CBM_), and bandgap (*E*_g_) of the SnO_2_ nanoparticles were − 4.36, − 4.31, and 3.79 eV, respectively [44]. Therefore, the *E*_CBM_ values of SnO_2_-cPCN can be calculated to be − 4.29 eV considering that the bandgap (~ 3.79 eV) of SnO_2_ films is not changed with or without cPCN. According to the theory study in literature [31], we reasonably speculate that the fully conjugated structure of cPCN changes the surface electron density of SnO_2_, resulting in such shift in the energy band of SnO_2_. Subsequently, the *E*_CBM_ values of perovskite are deduced using the same method to be − 4.27 eV with an *E*_g_ of 1.58 eV (Fig. S4b) [6]. The energy band diagram for a typical n-i-p PSC device (Fig. 2d) indicates that the SnO_2_-cPCN ETL is more favorable as an effective electron extraction channel conducive to reducing *V*_OC_ loss.

To ascertain the change in film morphology of ETL after hybridization, we show atomic force microscopy (AFM) and scanning electron microscope (SEM) images of the SnO_2_ and SnO_2_-cPCN films deposited on the FTO substrates in Figs. 2e, f and S7, respectively. The SnO_2_-cPCN film turns out to have a similar root-mean-square (RMS) roughness (37.7 nm) as that of the control film (41 nm). In Fig. S5, the water contact angle/perovskite solution of the SnO_2_-cPCN film is measured as 41°/47°, which is much larger than that of the pristine SnO_2_ (33.5°/38°). Furthermore, the contact angle of both films also is obtained after UV-ozone treatment and show the same trend (Fig. S6). According to the previous report [25], the non-wetting under-layer may lead to the formation of perovskite films with high-aspect-ratio crystalline grains since the lessened dragging force can result in high grain-boundary mobility [45]. Besides, Deng et al. further proposed that the attraction between the solute ions and solvent molecules on the hydrophilic surface was comparably stronger than the hydrophobic surface [46]. The strong attraction of the surface to the pre-existing clusters will fix more clusters on the substrate surface and block the re-dissolution of ions from pre-existing cluster surfaces. So, the existing cluster becomes more stable and easier to grow into crystal nuclei. In addition, the reaction heat released during cluster growth can be dissipated more rapidly through the surfaces due to a relatively higher interaction intensity, which is beneficial to the formation of the nucleus. Meanwhile, the precursor ions and solvent that are close to the surface will be attracted and then captured by the hydrophilic surface, which will slow down the diffusion of ions, leading to a slower crystal growth rate and smaller grain size [46–48]. Conversely, the non-wetting (hydrophobic) substrates can provide a higher free-energy barrier for nucleation, faster crystal growth rate, larger grain size, and less grain boundary [49, 50].

### Fabrication and Characterization of Perovskite Solar Cells

#### Perovskite Film Characterization

**Fig. 3:**
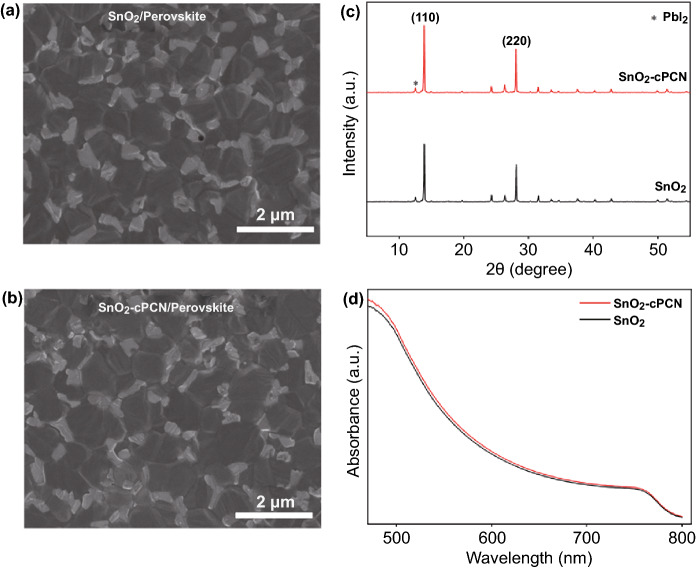
Top view SEM images of perovskite films coated on **a** SnO_2_ and **b** SnO_2_-cPCN substrates. **c** XRD patterns and **d** UV–Vis spectra of the perovskite films on SnO_2_ and SnO_2_-cPCN

Generally, the quality of perovskite films can be defined by some conspicuous features, such as grain size, crystallinity, and surface coverage, which collectively affect the performance of perovskite solar cells. Specifically, the topographic SEM images of perovskite films (prepared with the same composition and process) deposited on SnO_2_ and SnO_2_-cPCN are exhibited in Fig. 3a, b to investigate the influence of SnO_2_-cPCN hybridization on the morphology and crystallization of the perovskite films atop. Both films present dense and uniform morphology, with randomly interconnected grains. The statistical size distributions of perovskite grain clusters on different ETLs are plotted in Fig. S8. The perovskites deposited on SnO_2_ possessed an average grain size of about 1.08 µm. As the cPCN is incorporated into the SnO_2_, the appearance of pinholes decreased, and the average grain size significantly increased to 1.66 µm. These phenomena validated that more non-wetting surface after introducing cPCN did enlarge the grain size and decrease the grain boundaries of perovskite, in accord with the contact angle test.

To further study the effect of the cPCN incorporation on the solid structure of perovskite films, we collected XRD patterns of the perovskite films on two kinds of ETLs. It can be observed in Fig. 3c that both samples present a cubic perovskite phase structure, in which the prominent peaks around 13.98° and 28.22° correspond to the (110) and (220) planes. The diffraction peak at 12.6° in all films belongs to the PbI_2_ phase [45]. When cPCN was added, the intensity of (110) and (220) diffractions are enhanced, and the calculated full width at half maximum (FWHM) of (110) diffraction becomes smaller (0.112°) compared with the pristine sample (0.129°), suggesting a better-grown film with improved crystallinity. Subsequently, the UV–visible absorption spectra of the perovskite film grown on SnO_2_-cPCN further demonstrates slightly higher absorbance than that of the pristine film (Fig. 3d), contributing to enhancing photocurrents in the PSCs (vide infra).

#### Photovoltaic Performance

**Fig. 4:**
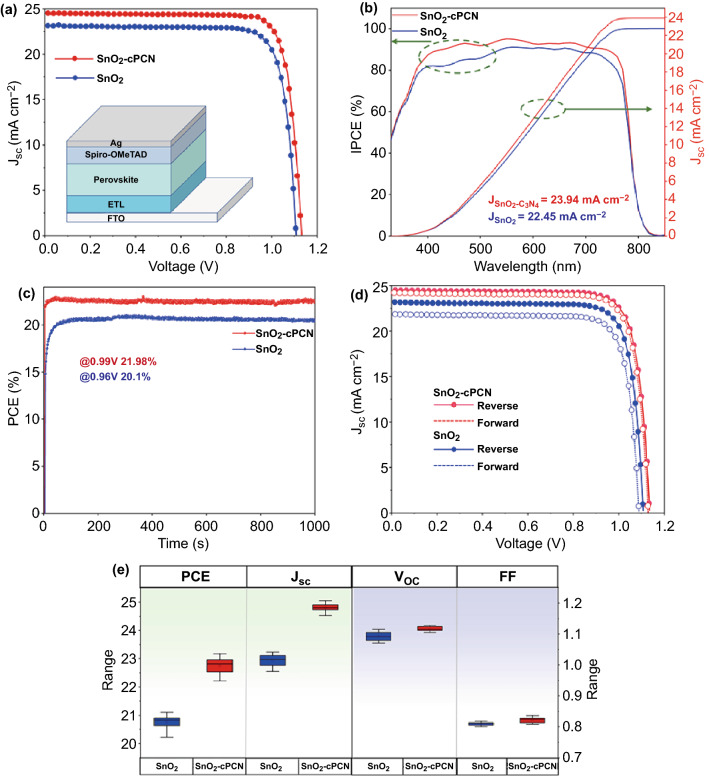
**a** *J–V* characteristics for champion PSCs based on the SnO_2_ and SnO_2_-cPCN ETLs under the illumination of 1 sun (AM 1.5 G). **b** Corresponding EQE curves and integrated current density of the two champion PSCs. **c** The stabilized power output of the fabricated PSCs on the SnO_2_-cPCN and SnO_2_ ETL at the maximum power point (MPP) at 0.99 and 0.96 V. **d** *J–V* curves of both champion devices for both forward and reverse scans. **e** Distribution of photovoltaic parameters of the two kinds of solar cells (20 devices for each case)

The photovoltaic performance of the novel SnO_2_-cPCN ETL based PSCs was examined by fabricating a series of (FAPbI_3_)_0.9_(MAPbBr_3_)_0.1_-based planar-type PSCs with a structure of FTO/ETL/perovskite/Spiro-OMeTAD/Ag, as exhibited in the inset of Fig. 4a. The optimization results of cPCN concentrations in SnO_2_ colloidal are provided in Fig. S9 and Table S1. Besides, the current density versus voltage (*J–V*) characteristics for the champion PSCs based on the SnO_2_ and SnO_2_-cPCN ETLs under AM 1.5G illumination (100 mW cm^−2^) are illustrated in Fig. 4a. The main cell parameters of *V*_OC_, *J*_SC_, *FF*, and *PCE* are summarized in Table 1. The device based on the pristine SnO_2_ substrate presentes a PCE of 21.3% with *J*_SC_ of 23.4 mA cm^−2^, *V*_OC_ of 1.11 V, and *FF* of 82%. After optimization, the best device (0.1cm^2^) is obtained with a *PCE* of 23.17%, a *V*_OC_ as high as 1.126 V, a *J*_SC_ of 24.9 mA cm^−2^, and *FF* of 82.5%. It is one of the best performances in PSCs with modified SnO_2_ [15, 51, 52]. The superior performance of PSCs with cPCN-treated SnO_2_ is in line with the improved film quality and higher absorptions. In Fig. 4b, the incident photon-to-current efficiencies (IPCE) of the optimal solar cells based on SnO_2_-cPCN show significant enhancement over 400–770 nm wavelength than that based on unmodified SnO_2_, which can be attributed to the enhanced UV–visible absorption spectra of the perovskite film grown on SnO_2_-cPCN with improved crystallinity (Fig. 3c). The integrated *J*_SC_ of 23.94 and 22.45 mA cm^−2^ for PSCs with SnO_2_-cPCN and SnO_2_ can be calculated and in acceptable agreement with the *J–V* results. The increase of *J*_SC_ mainly originates from the improved band alignment, better carrier transport from perovskite to ETLs, and enhanced light absorbance. The stabilized power output (SPO) of PSCs was performed to determine the power output stability of the devices at the maximum power point (MPP) under AM 1.5G illumination (Fig. 4c). The output PCE of the sample with SnO_2_-cPCN as the ETL is traced for 1000 s at MPP (0.99 V), and a stabilized *PCE* of 21.98% can be recorded, matching the PCE value extracted from the *J–V* curve. For the control device, a stabilized PCE of 20.1% at the maximum power point (0.96 V) is recorded over the same period. Notably, the SnO_2_-cPCN based device has a much faster response of less than 8 s to reach the SPO point than the pristine device (80 s) ascribed to the reduced trap-assisted recombination and the enhancement of electron mobility caused by the incorporation of cPCN [53].

**Table 1:** Device performance of champion PSCs based on SnO_2_ and SnO_2_-cPCN ETLs

ETLs	Area (cm^2^)	Scan direction	*V*_OC_ (V)	*J*_SC_ (mAcm^−2^)	*FF* (%)	*PCE* (%)	*HI* (%)
SnO_2_	0.1	Reverse	1.11	23.4	82	21.3	6.5
		Forward	1.1	22.4	80.5	19.9	
SnO_2_-cPCN	0.1	Reverse	1.126	24.9	82.5	23.17	1.5
		Forward	1.125	24.7	82	22.8	
	1	Reverse	1.13	24	75	20.3	0.4
		Forward	1.12	23.9	75.5	20.2	

Ion migration, high trap density, and unbalanced charge transport are currently considered the main reasons for the hysteresis [54, 55]. To investigate the hysteresis effect in our system, we measured *J-V* curves along with both bias scanning directions in Fig. 4d and Table 1. The hysteresis indices (H_hysteresis_) of the devices were calculated based on Eq. 1 [56]. Besides, the device using SnO_2_-cPCN ETL shows negligible hysteresis, contrasting to the much larger effect with the SnO_2_ ETL. The crucial role of SnO_2_-cPCN in resolving the hysteresis issue was also checked with transient photocurrent decay (TPC) and photovoltage decay (TPV) (Fig. S10a, b). It is revealed that the time constant of the photocurrent decay significantly decreases while the decay time of the photovoltage increases when the device was fabricated based on SnO_2_-cPCN ETL. Due to the comparable electron mobility of SnO_2_-cPCN ETL (3.3 × 10^–3^ cm^2^ V^−1^ s^−1^) to the hole mobility of the doped spiro-OMeTAD (~ 10^–3^ cm^2^ V^−1^ s^−1^), the electron flux (Fe) is essentially equal to the hole flux (Fh) considering that the interface areas of both ETL/perovskite and perovskite/HTL are the same. Therefore, charge accumulation on either side of the devices based on the SnO_2_-cPCN can be alleviated, which, consequently, exhibit negligible hysteresis (Fig. S11) [19, 57]. In addition, we speculate that the K^+^ in carbon nitride may also contribute to the reduced hysteresis in the devices [58, 59]. The statistical analysis of all parameters (*V*_OC_, *J*_SC_, *FF*, and *PCE*) of PSCs based on SnO_2_ and SnO_2_-cPCN are provided in Fig. 4e (20 cells counted for each case). Fascinatingly, the devices based on SnO_2_-cPCN exhibite excellent repeatability with a minimal standard deviation and reliability in contrast to the devices based on unmodified SnO_2_.

#### Charge transfer dynamics

**Fig. 5:**
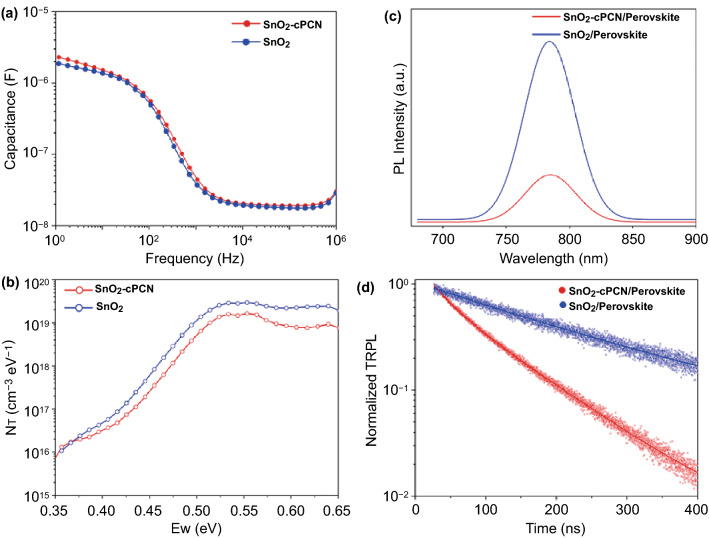
**a** Frequency-capacitance measured from perovskite devices on the SnO_2_-cPCN and SnO_2_ ETL. **b** Trap density of states (tDOS) for devices with SnO_2_-cPCN and SnO_2_ ETL. **c** Steady-state PL and **d** TRPL spectra of perovskite films deposited on different substrates

Further insights into the origin of the performance enhancement for the device using SnO_2_-cPCN ETL can be obtained from the thermal admittance spectroscopy (TAS) measurement by examining the trap density of states (tDOS) in the devices with and without cPCN (Fig. 5a). TAS is a well-established, effective technique for characterizing both shallow and deep defects and has been broadly adopted to understand defects in the thin film [60, 61]. The defect density (*N*_*T*_) can be estimated by Eq. 3:

3 $$N_{T} \left( {E_{\omega } } \right) = - \frac{{V_{bi} }}{qW}\frac{{{\text{d}}C}}{{{\text{d}}\omega }}\frac{\omega }{{TK_{B} }}$$

where *C* is the capacitance, *ω* is the angular frequency, *q* is the elementary charge, *k*_*B*_ is the Boltzmann’s constant, and *T* is the temperature. *V*_bi_ and W represent the built-in potential and depletion width, respectively, and can be obtained from the previous report [62]. The applied angular frequency *ω* defines the energetic demarcation,

4 $$E_{\omega } = K_{B} T\ln \left( {\frac{{\omega_{0} }}{\omega }} \right),$$

where *ω*_0_ denotes the attempt-to-escape frequency and can be output by frequency-dependent capacitance plot [63]. As illustrated in Fig. 5b, the devices with SnO_2_-cPCN ETL have dramatically lower trap density than the SnO_2_ ETL (above 0.4 eV). This demonstrates that the perovskite grown on SnO_2_-cPCN ETL possesses reduced deep-level defects at the grain boundary due to the enhanced grain size, contributing to reducing the recombination of carriers and enhancing the performance of PSCs device dramatically [64, 65]. Furthermore, the electron-only devices with the structure of FTO/ETL/Perovskite/PCBM/Ag were also manufactured for space charge limited current (SCLC) measurement as circumstantial evidence to the decreased trap density of perovskite deposited on SnO_2_-cPCN. The dark current–voltage (*I–V*) curves are illustrated in Fig. S12a, b. The trap densities of the perovskite film coated on SnO_2_ and SnO_2_-cPCN substrates are 1.75 × 10^16^ and 8.39 × 10^15^ cm^−3^, respectively. The reduced trap density is consistent with TAS measurement.

The charge carrier recombination dynamics of the perovskites deposited on different ETLs were investigated by steady-state photoluminescence (PL) and time-resolved photoluminescence (TRPL) decay measurements. The characteristic PL spectra of all the perovskite films are located at around 784 nm (Fig. 5c). Compared with the pristine sample, the PL intensity of SnO_2_-cPCN based perovskite films is much weaker, demonstrating enhanced charge extraction with reduced recombination, leading to increased photocurrents in the PSCs [53]. For the TRPL spectra in Fig. 5d, all TRPL curves are fitted by a biexponential function as follows:

5 $$y = A_{1} \times e^{{ - t/\tau_{1} }} + A_{2} \times e^{{ - t/\tau_{2} }} + A_{0},$$

where *A*_1_ and *A*_2_ denote the relative amplitude fractions for *τ*_1_ and *τ*_2_, respectively. Generally, the fast decay lifetime (*τ*_1_) results from the trap-assisted recombination at the interface, and the slow decay lifetime (*τ*_2_) is related to the biomolecular recombination of photogenerated free carriers due to traps in bulk [66, 67]. The fast decay component (*τ*_1_) for perovskite film deposited on the SnO_2_ layer is 176 ns. After cPCN is incorporated into the SnO_2_ layer, *τ*_1_ is shortened to 30 ns, confirming the much faster charge extraction and transport through the SnO_2_-cPCN compared to the bare SnO_2_. Besides, the reduced interfacial recombination was correlated with the improved conductivity in SnO_2_-cPCN ETL, preventing charge accumulation at the perovskite/ETLs interface [68]. A decrease in recombination at the perovskite/ETLs interface is conducive to promoting the *V*_*OC*_ and *J*_*SC*_, resulting from the decreased trap density in ETLs. The result is highly consistent with PL and SCLC measurements.

**Fig. 6:**
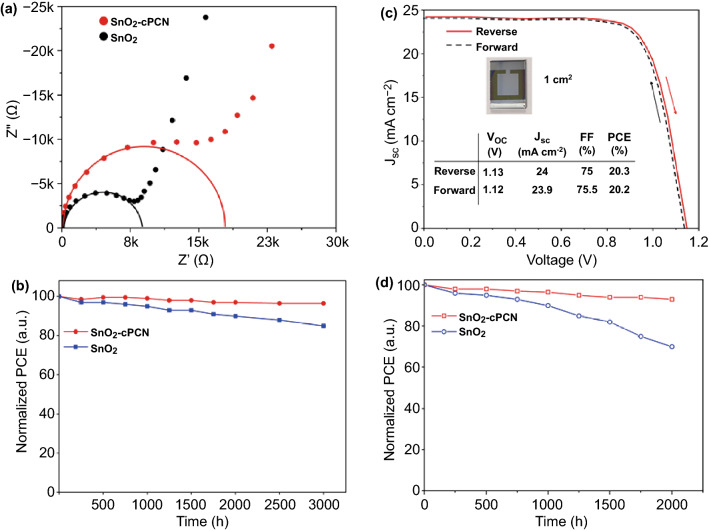
**a** EIS of planar-type PSCs with SnO_2_ and SnO_2_-cPCN ETL, the insert picture is the fitting model. **b** *J–V* characteristics of the SnO_2_-cPCN and SnO_2_ based devices with a 1 cm^2^ area (active area) in forward and reverse scan; the inset is a picture of real 1 cm^2^ area devices. **c** Long-term stability measurements of devices without any encapsulation under N_2_ glovebox, and **d** Humidity and oxygen stability under an ambient condition (relative humidity: 30% ± 5%)

Electrochemical impedance spectroscopy (EIS) was conducted to investigate the charge transport and recombination behavior. The semicircles in the low and high-frequency regions are attributed to the recombination resistance (*R*_rec_) and the transfer resistance (*R*_tr_), respectively [69]. Figure 6a exhibits the Nyquist plots of the control and optimal devices in the dark with a bias of 0.5 V and a frequency from 0.1–10^5^ Hz. It is revealed that the devices with the SnO_2_-cPCN ETL have larger *R*_rec_ than the control device owing to the reduced surface/interface trap states and other recombination centers [70]. This behavior agrees with the increased carrier lifetime in TRPL and enhanced V_OC_ and J_SC_ of PSCs based on SnO_2_-cPCN ETL. Since the SnO_2_-cPCN-based devices presented outstanding performance with a small active area (0.1 cm^2^), the large-area (active area, 1 cm^2^) devices were further fabricated to assess the authenticity of cPCN in ETL of PSCs. The *J–V* curves measured from both directions are illustrated in Fig. 6b, and the inset shows the image of our large area device. The device generates a *V*_OC_ of 1.13 V, a *J*_SC_ of 24 mA cm^−2^, an *FF* of 75%, and a *PCE* of 20.3% in the reverse scan. Similarly, the hysteresis of the devices is visibly reduced when cPCN was added to SnO_2_ in large-area devices. Our previous characterizations have demonstrated the increased electron mobility with SnO_2_-cPCN and the decreased trap density of the perovskite film deposited on SnO_2_-cPCN, forming the primary reasons for reduced hysteresis.

#### Stability Test

Stability tests are a pivotal characterization of the PSCs. The long-term stability of unencapsulated PSCs fabricated with or without cPCN hybridization was investigated with the devices stored under dark in a glove box filled with nitrogen. As indicated in Fig. 6c, the device based on SnO_2_-cPCN ETL maintains 95% of the original efficiency after 2880 h (~ 4 months). However, the device based on SnO_2_ only keeps 85% of its initial efficiency under the same storage condition. Regarding the stability of PSCs under humidity and oxygen, the SnO_2_-cPCN-based device also presents higher stability than SnO_2_-based devices, maintaining 88% of the initial PCE after 2000 h of storage in the ambient environment (with controlled RH of 30% ± 5%) without any encapsulation (Figs. 6d and S13). For the same test duration, SnO_2_-based devices remains only 64% relative to their initial efficiency. Apparently, the stability against oxygen, humidity, and long-term stability has been significantly improved with the increased quality of the perovskite film deposited on SnO_2_-cPCN. The enhancement of stability can be attributed to the suppressed charge accumulation on the photoexcited perovskite and superoxide-mediated degradation pathway under the increased electron transfer with the SnO_2_-cPCN ETL [71].

## Conclusion

To sum up, a novel and effective SnO_2_-cPCN composite yielding superior electron mobility of 3.3 × 10^−3^ cm^2^ V^−1^ s^−1^ was produced. It is more than 3 times higher than that of neat SnO_2_. Notably, the champion PCEs of the planar PSCs based on SnO_2_-cPCN reached 23.17% on a small area device (0.1 cm^2^), and the PCE of 20.3% was obtained on a 1 cm^2^ device. The improved performance of the PSCs based on SnO_2_-cPCN can be attributed to the following advantages. First, the surface wettability of SnO_2_-cPCN slightly decreased after the cPCN addition, suppressing heterogeneous nucleation and enlarging perovskite grain size. Consequently, high-quality perovskite films with reduced grain boundaries and mitigated non-radiative recombination were generated. Second, the high electron mobility and improved band alignment of SnO_2_-cPCN ETL reduced the charge accumulation at the perovskite/ETL interface, leading to negligible current density–voltage hysteresis. This work provides a promising direction for developing high-quality ETLs and verifies the enormous potential of large-scale deployment of perovskite photovoltaics.

## Supplementary Information

Below is the link to the electronic supplementary material.

Supplementary file1 (DOCX 2554 kb)
