# Supplementary material for: cPCN-Regulated SnO2 Composites Enables Perovskite Solar Cell with Efficiency Beyond 23%
Source: Nanomicro Lett. 2021 Apr 1;13:101. doi: 10.1007/s40820-021-00636-0 (PMC8017043; doi:10.1007/s40820-021-00636-0)
Supplement: Supplementary file 1 — Supplementary file1 (DOCX 2554 kb) [file 40820_2021_636_MOESM1_ESM.docx]

Supporting Information for

**cPCN-Regulated SnO_2_ Composites Enables Perovskite Solar Cell with Efficiency Beyond 23%**

Zicheng Li^1, 2, 3^, Yifeng Gao^1, 3^, Zhihao Zhang^1, 2, 3^, Qiu Xiong^1, 3^, Longhui Deng^1, 3^, Xiaochun Li^2^, Qin Zhou^1, 3^, Yuanxing Fang^2^, Peng Gao^1, 3,^ *

^1^CAS Key Laboratory of Design and Assembly of Functional Nanostructures, and Fujian Provincial Key Laboratory of Nanomaterials Fujian Institute of Research on the Structure of Matter, Chinese Academy of Sciences, Fuzhou, Fujian 350002, People’s Republic of China

^2^College of Chemistry, Fuzhou University, Fuzhou 350116, People’s Republic of China

^3^Laboratory for Advanced Functional Materials, Xiamen Institute of Rare Earth Materials, Haixi Institute, Chinese Academy of Sciences, Xiamen 361021, People’s Republic of China

*Corresponding author. E-mail: [peng.gao@fjirsm.ac.cn](mailto:peng.gao@fjirsm.ac.cn) (Peng Gao)

**Supplementary Figures and Tables**


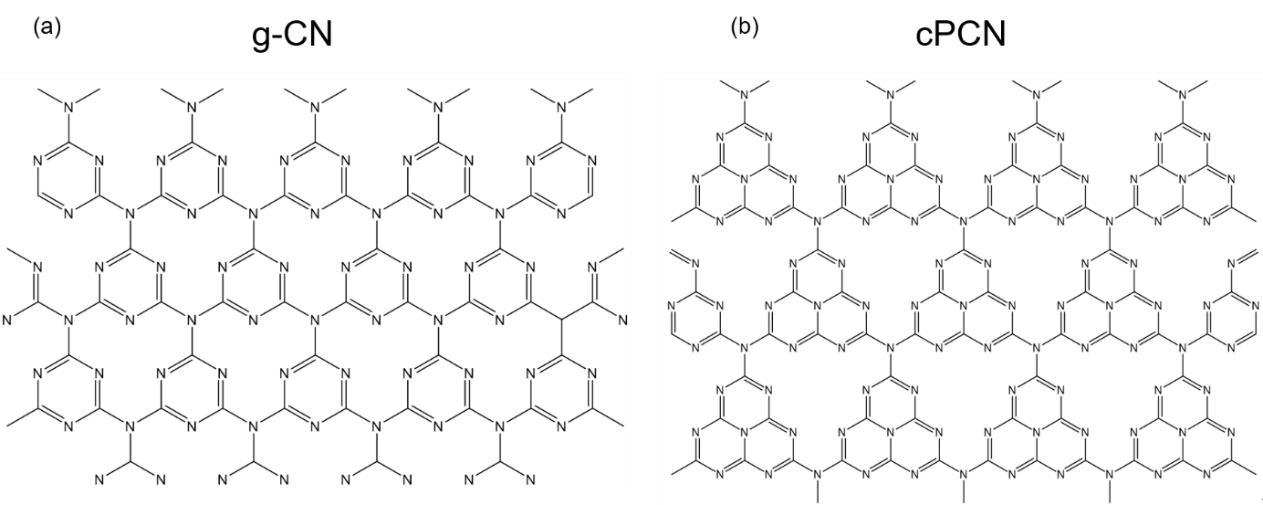


**Fig. S1** Structure models of g-CN, cPCN


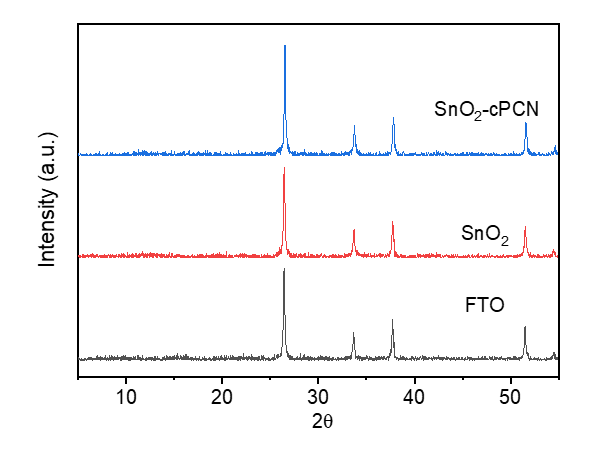


**Fig. S2** XRD spectra of FTO, SnO_2_ and SnO_2_-cPCN


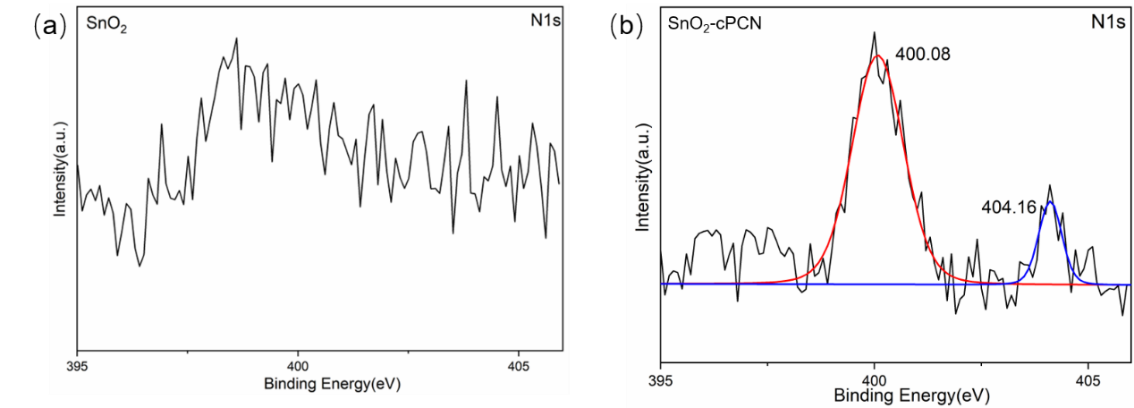


**Fig. S3** XPS spectra of N 1s of **(a)** SnO_2_ and **(b)** SnO_2_-cPCN


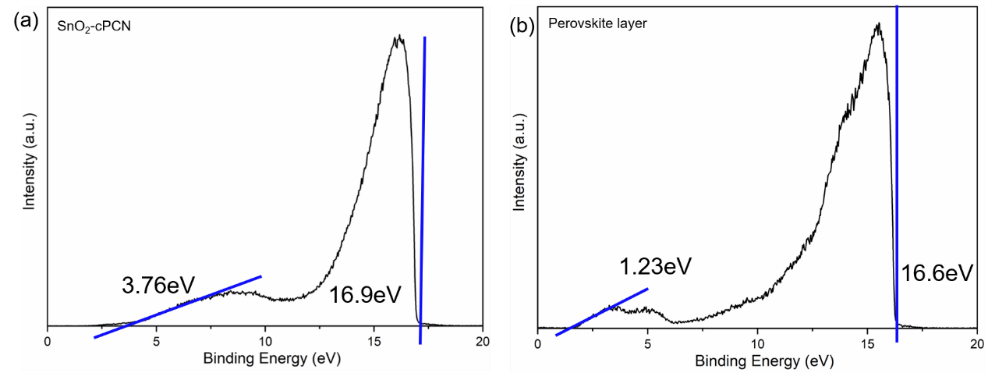


**Fig. S4** UPS spectra for SnO_2_-cPCN film and perovskite film deposited on glass


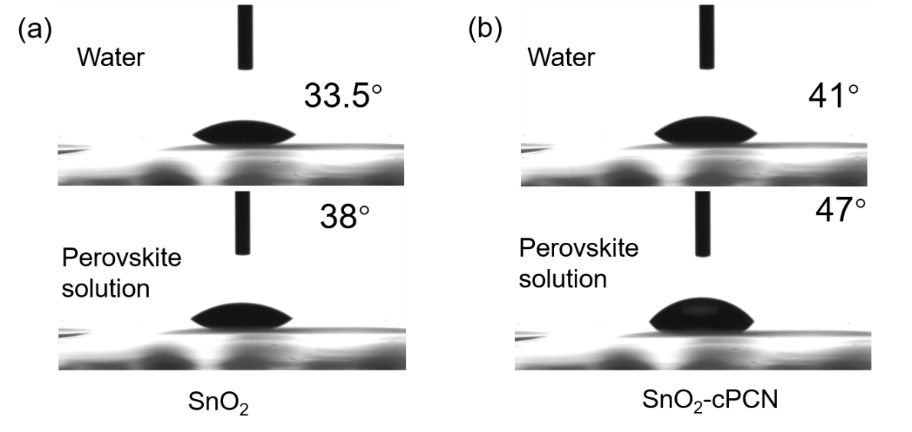


**Fig. S5** Contact angle measurements of water and perovskite solution on SnO_2_ and SnO_2_-cPCN films


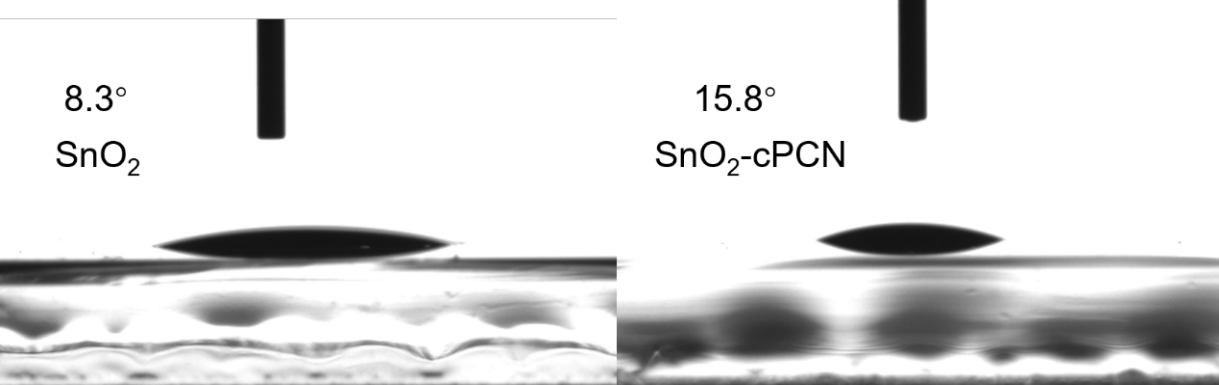


**Fig. S6** Contact angle measurements of perovskite solution on SnO_2_ and SnO_2_-cPCN films after UV-ozone treatment


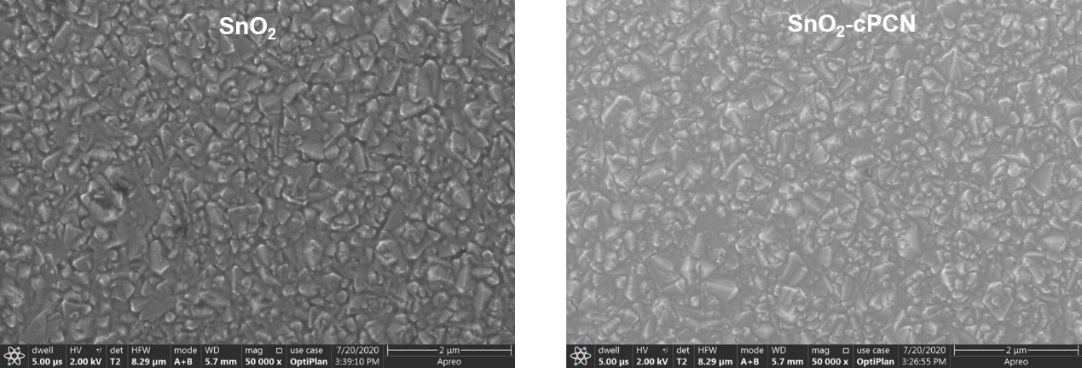


**Fig. S7** SEM images of SnO_2_ **(**Left**)** and SnO_2_-cPCN film **(**Right**)**


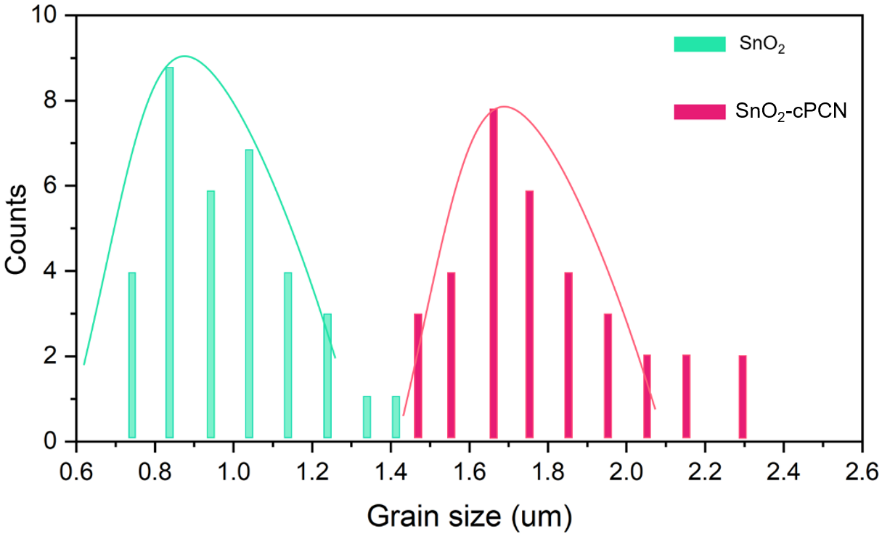


**Fig. S8** Average grain size of the perovskite films on SnO_2_ and SnO_2_-cPCN derived from Fig 3a, b


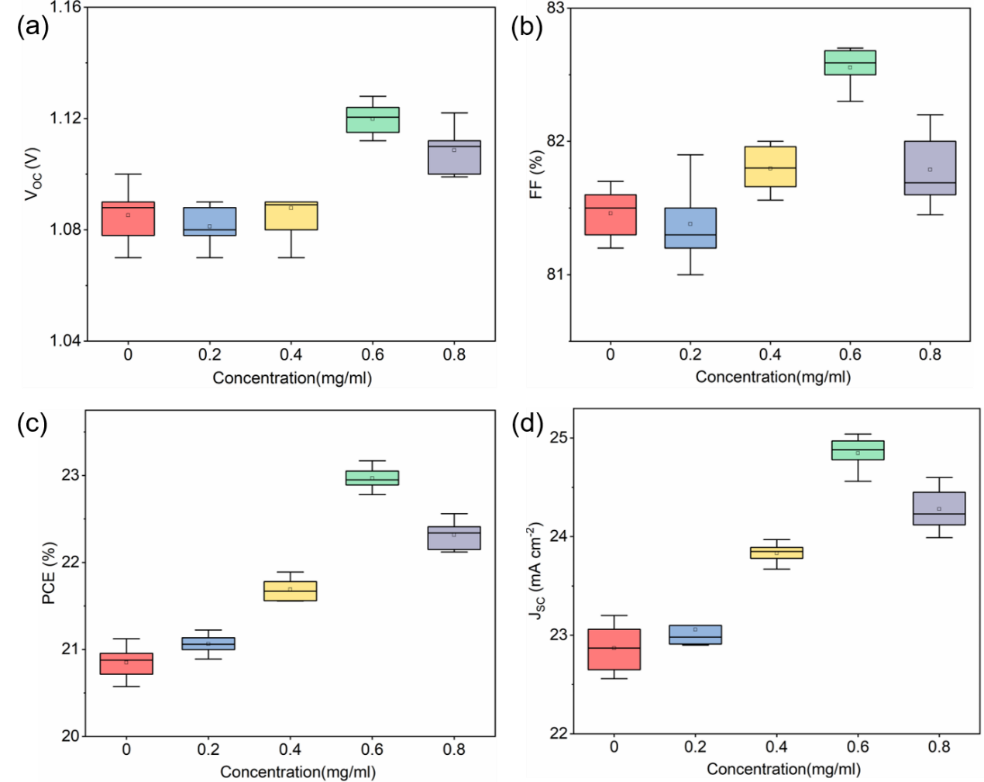


**Fig. S9** **(a-d**) PCE, FF, V_OC_, and J_SC_ extracted from their corresponding J-V curves as functions of the cPCN concentration in the SnO_2_ solution for the preparation of SnO_2_-cPCN ETL


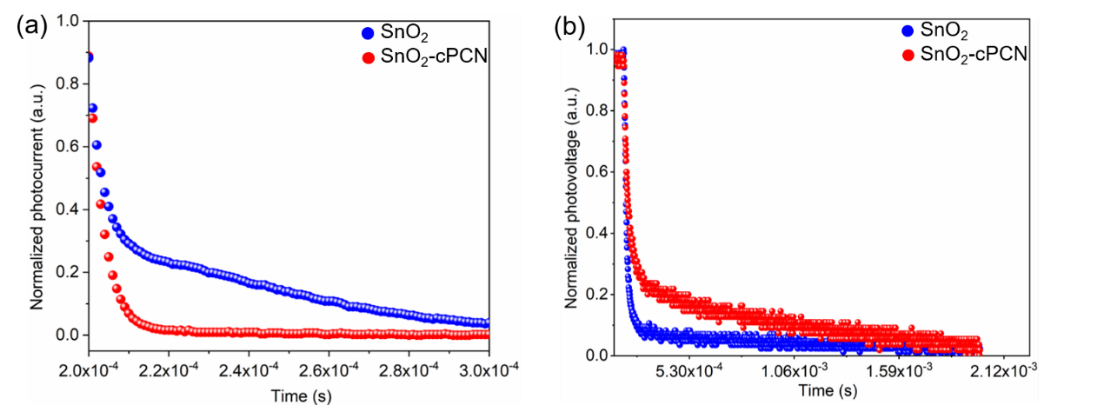


**Fig. S10** (**a**) Normalized transient photocurrent decay and (**b**) normalized transient photovoltage decay of PSCs with SnO_2_ and SnO_2_-cPCN as ETL


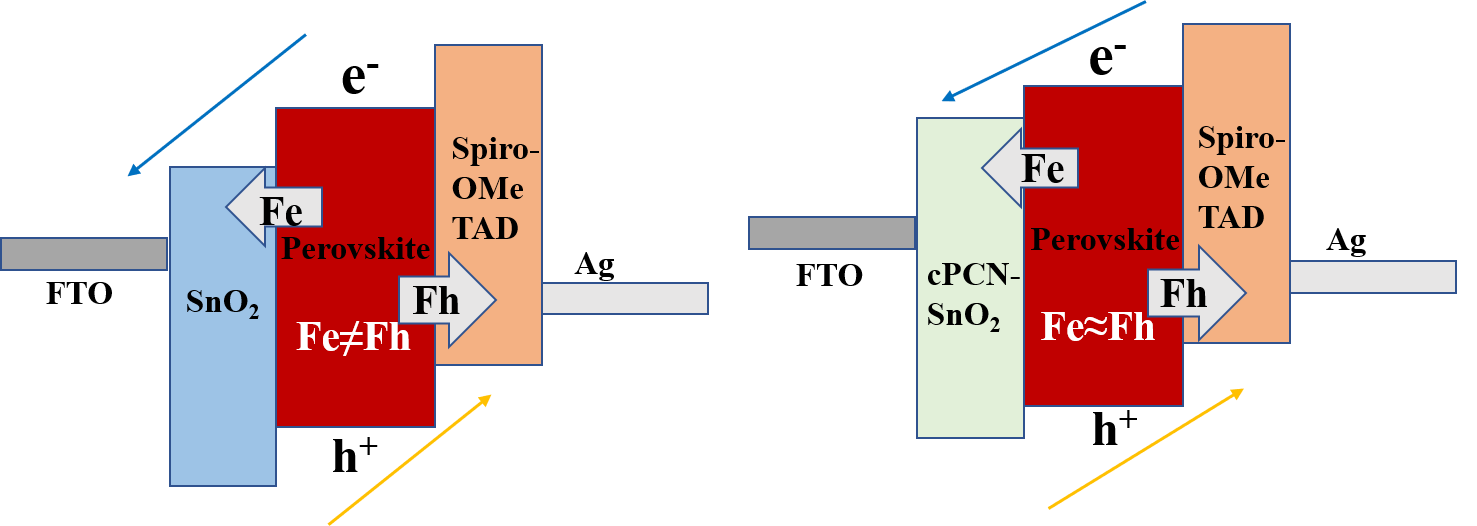


**Fig. S11** Charge transport mechanism. (a) Planar-type PSCs with SnO_2_ and (b) SnO_2_-cPCN


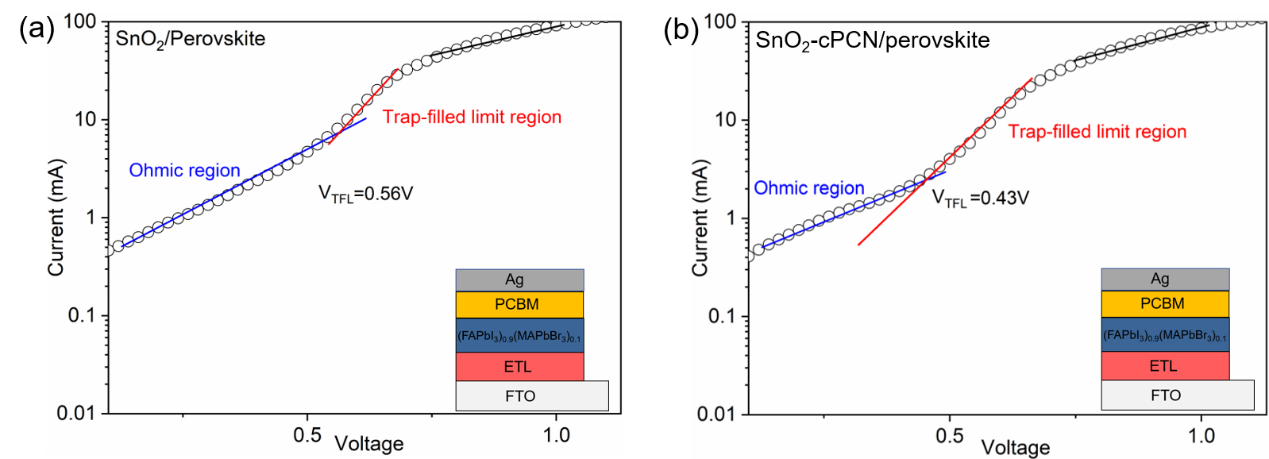


**Fig. S12** Dark I–V curves of the electron-only devices with the V_TFL_ kink points. The inset shows the structure of the electron-only device


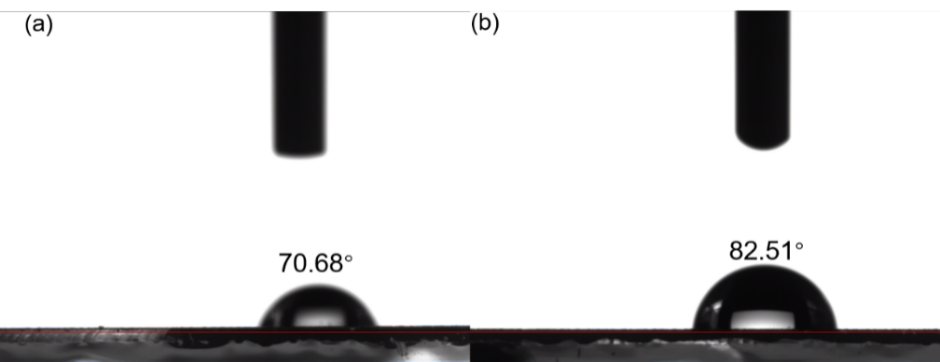


**Fig. S13** Contact angle of perovskite film based on (**a**) SnO_2_ and (**b**) SnO_2_-cPCN

**Table S1** Device performance of V_OC_, J_SC_, FF, and PCE of devices at different cPCN concentrations

| Concentration(mg/ml) | Voc (V) | J_SC_ (mAcm^−2^) | FF (%) | PCE (%) |
| --- | --- | --- | --- | --- |
| 0 | 1.11 | 23.2 | 82 | 21 |
| 0.2 | 1.11 | 23.5 | 81.5 | 21.5 |
| 0.4 | 1.115 | 23.9 | 82 | 21.75 |
| 0.6  0.8 | 1.126  1.119 | 24.9  24.3 | 82.5  81.8 | 23.17  22.24 |

**Table S2** Summary of the modified SnO_2_ based perovskite solar cells

| ETL Type | μ_e_ (SnO_2_) | μ_e_ (modified SnO_2_) | Perovskite type | Year | PCE | Refs. |
| --- | --- | --- | --- | --- | --- | --- |
| EDTA- SnO_2_ | 9.92 × 10^−4^ | 2.27 × 10^−3^ | FACsI | 2018.8 | 21.6 | [S1] |
| Nb-SnO_2_ | 1.02 × 10^−4^ | 2.16 × 10^−4^ | FAMAPbIBr | 2016.12 | 17.57 | [S2] |
| SnO_2_–HP | 1.52 × 10^−3^ | 2.76× 10^−3^ | CsFAMAPbIBr | 2020.7 | 23.06 | [S3] |
| SnOx: NdCl_3_ | 1.46 × 10^-3^ | 6.29 × 10^-3^ | FA_1-x_MA_x_PbI_3_ | 2020.9 | 21.49 | [S4] |
| SnO_2_:GQDs | 6.72 × 10^−4^ | 1.01 × 10^−3^ | MAPbI_3_ | 2017.8 | 20.31 | [S5] |
| SnO_2_-RCQs | 9.32 × 10^−4^ | 1.73 × 10^−2^ | CsFAMAPbIBr | 2019.11 | 22.77 | [S6] |
| G- SnO_2_ | 5.2 × 10^−3^ | 7.5× 10^−3^ | CsFAMAPbIBr | 2019.12 | 22.13 | [S7] |
| S-SnO_2_ | 3.37 × 10^-4^ | 3.46 × 10^-3^ | FAMAPbIBr | 2020.9 | 22.84 |  |
| Nd- SnO_2_ | 12.1×10^-4^ | 36.1×10^-4^ | CsFAMAPbIBr | 2020.6 | 20.92 | [S8] |
| **cPCN-SnO_2_** | **9.95×10^-4^** | **3.3×10^-3^** | **FAMAPbIBr** | **2020.12** | **23.17** | **This work** |

**Supplementary References**

[S1] D. Yang, R. Yang, K. Wang, C. Wu, X. Zhu et al., High efficiency planar-type perovskite solar cells with negligible hysteresis using EDTA-complexed SnO_2_ Nat. Commun. **9**(1), 3239 (2018). <https://doi.org/10.1038/s41467-018-05760-x>

[S2] X. Ren, D. Yang, Z. Yang, J. Feng, X. Zhu et al., Solution-processed Nb: SnO_2_ electron transport layer for efficient planar perovskite solar cells. ACS Appl. Mater. Interfaces **9**(3), 2421-2429 (2017). <https://doi.org/10.1021/acsami.6b13362>

[S3] S. You, H. Zeng, Z. Ku, X. Wang, Z. Wang et al., Multifunctional polymer-regulated SnO_2_ nanocrystals enhance interface contact for efficient and stable planar perovskite solar cells. Adv. Mater. **32**(43), e2003990 (2020). <https://doi.org/10.1002/adma.202003990>

[S4] Q. Xiong, L. Yang, Q. Zhou, T. Wu, C. L. Mai et al., NdCl_3_ dose as a universal approach for high-efficiency perovskite solar cells based on low-temperature-processed snox. ACS Appl. Mater. Interfaces **12**(41), 46306-46316 (2020). <https://doi.org/10.1021/acsami.0c13296>

[S5] J. Xie, K. Huang, X. Yu, Z. Yang, K. Xiao et al., Enhanced electronic properties of SnO_2_ via electron transfer from graphene quantum dots for efficient perovskite solar cells. ACS Nano **11**(9), 9176-9182 (2017). <https://doi.org/10.1021/acsnano.7b04070>

[S6] W. Hui, Y. Yang, Q. Xu, H. Gu, S. Feng et al., Red-carbon-quantum-dot-doped SnO_2_ composite with enhanced electron mobility for efficient and stable perovskite solar cells. Adv. Mater. **32**(4), e1906374 (2020). <https://doi.org/10.1002/adma.201906374>

[S6] J. Chen, H. Dong, L. Zhang, J. Li, F. Jia et al., Graphitic carbon nitride doped SnO_2_ enabling efficient perovskite solar cells with pces exceeding 22%. J. Mater. Chem. A **8**(5), 2644-2653 (2020). <https://doi.org/10.1039/c9ta11344d>

[S7] J. Jia, J. Dong, J. Wu, H. Wei, B. Cao, Combustion procedure deposited SnO_2_ electron transport layers for high efficient perovskite solar cells. J. Alloys Compd. **844** 156032 (2020). <https://doi.org/10.1016/j.jallcom.2020.156032>
